# Altering textural properties of fermented milk by using surface‐engineered *Lactococcus lactis*

**DOI:** 10.1111/1751-7915.13278

**Published:** 2018-05-09

**Authors:** Mariya Tarazanova, Thom Huppertz, Jan Kok, Herwig Bachmann

**Affiliations:** ^1^ NIZO B.V. P.O. Box 20 6710 BA Ede The Netherlands; ^2^ TiFN P.O. Box 557 6700 AN Wageningen The Netherlands; ^3^ Molecular Genetics University of Groningen Nijenborgh 7 9747AG Groningen The Netherlands; ^4^ Present address: FrieslandCampina Stationsplein 4 3818 LE Amersfoort The Netherlands

## Abstract

Lactic acid bacteria are widely used for the fermentation of dairy products. While bacterial acidification rates, proteolytic activity and the production of exopolysaccharides are known to influence textural properties of fermented milk products, little is known about the role of the microbial surface on microbe–matrix interactions in dairy products. To investigate how alterations of the bacterial cell surface affect fermented milk properties, 25 isogenic *Lactococcus lactis* strains that differed with respect to surface charge, hydrophobicity, cell chaining, cell‐clumping, attachment to milk proteins, pili expression and EPS production were used to produce fermented milk. We show that overexpression of pili increases surface hydrophobicity of various strains from 3–19% to 94–99%. A profound effect of different cell surface properties was an altered spatial distribution of the cells in the fermented product. Aggregated cells tightly fill the cavities of the protein matrix, while chaining cells seem to be localized randomly. A positive correlation was found between pili overexpression and viscosity and gel hardness of fermented milk. Gel hardness also positively correlated with clumping of cells in the fermented milk. Viscosity of fermented milk was also higher when it was produced with cells with a chaining phenotype or with cells that overexpress exopolysaccharides. Our results show that alteration of cell surface morphology affects textural parameters of fermented milk and cell localization in the product. This is indicative of a cell surface‐dependent potential of bacterial cells as structure elements in fermented foods.

## Introduction

Lactic acid bacteria (LAB) are Gram‐positive bacteria that are generally regarded as safe (GRAS) and are used extensively in food and feed fermentations. They are also found on mucosal surfaces of humans and animals (Stiles and Holzapfel, 1997; Leroy and De Vuyst, 2004). One of the dominant attributes of LAB is the fact that they produce lactic acid as the main metabolic end‐product of fermentation, which leads to acidification and preservation of the fermented product. An additional functionality of many strains is their ability to produce volatile metabolites that are important flavour compounds (Smit *et al*., 2004, 2005a,b). LAB can also play a significant role in altering textural properties of the end‐products through acidification, proteolytic activity or the production of extracellular polysaccharides (EPS; Smid and Kleerebezem, 2014). One economically very important species of LAB, *Lactococcus lactis*, is used worldwide in the dairy industry for the production of quark, buttermilk and a huge variety of cheeses.

In general, the matrix of fermented dairy products like yoghurt and cheese consists of aggregated caseins, whey proteins and fat droplets, interspersed with serum and/or whey pockets. In addition, it contains minerals, salts and microorganisms. Interactions between milk components in the matrix and their effect on functionality have been studied extensively (Morell *et al*., 2014; Dickinson, 2015; Hadde *et al*., 2015; Joyner and Damiano, 2015; Li and Nie, 2015). For example, apart from protein–protein interactions, the rheological properties of a milk gel also depend on the size and number of fat droplets and their surface composition (Mao and McClements, 2013). When milk is homogenized, the fat droplets are covered and stabilized by milk proteins. Because of the cross‐linking interactions between proteins covering the fat droplets, the milk gel is strong and has a low flow and enhanced stability against the expulsion of serum from the contracting protein matrix, syneresis. Interactions between constituents in the food matrix can be hydrophobic (Jarunglumlert *et al*., 2015), electrostatic (Cheng *et al*., 2015), based on hydrogen bonding (Wang *et al*., 2015), Van der Waals, depletion interaction (Tuinier *et al*., 2015), the consequence of steric repulsion (Evans *et al*., 2013) and/or caused by salt bridges (Bosshard *et al*., 2004).

Relative to the large body of knowledge on interactions between the various milk components (Jelen, 2005; Lucey *et al*., 2017), little is known about microbe–matrix interactions and the possible interactions between LAB and matrix components of fermented products (Busscher and Weerkamp, 1987; Ploux *et al*., 2010; Burgain *et al*., 2014a, 2015). Interactions between microorganisms and milk components could occur via surface properties of both particles (Ly‐Chatain *et al*., 2010; Burgain *et al*., 2014b), while different types of interactions can occur at the same time, such as Van der Waals interactions, electrostatic repulsions and attractions, hydrogen bonds, hydrophobic interactions, salt bridges and steric interactions (Ubbink and Schär‐Zammaretti, 2005; Jacobs *et al*., 2007; Burgain *et al*., 2013). The surface properties of bacteria are determined by the molecular composition of their cell walls, which can be decorated with (lipo‐)teichoic acids, proteins (Habimana *et al*., 2007), pili or capsular polysaccharides (CPS; Delcour *et al*., 1999; Giaouris *et al*., 2009; Meyrand *et al*., 2013).

The biodiversity of *L. lactis* surface properties is very high (Tarazanova *et al*., 2017). Pili can significantly change the surface of *L. lactis* upon their overexpression (Tarazanova *et al*., 2016). Large surface changes can also be introduced by plasmid transfer through conjugation (Neve *et al*., 1987; Broadbent and Kondo, 1991; Gasson *et al*., 1995), or by altering the expression of genes such as those encoding autolysins (Buist *et al*., 1997; Visweswaran *et al*., 2013) or enzymes involved in galactose utilization (Grossiord *et al*., 2003). The molecular composition of the cell wall has a big impact on the roughness of the bacterial surface, on bacterial chaining and on cell aggregation (Tarazanova *et al*., 2016). These properties govern the interactions between LAB and the matrix components (Ly‐Chatain *et al*., 2010; Burgain *et al*., 2014a). The production of EPS by a starter culture is known to affect textural properties through the binding of water and thereby increasing milk ropiness. This leads to an increase in milk viscosity and a reduction of syneresis (Hassan *et al*., 2003; Amatayakul *et al*., 2006; Prasanna *et al*., 2013). The charge, stiffness and linearity of EPS molecules impacts on rheological and physical properties of the fermented milk matrix. EPS modification by partial removal of side groups leads to a reduction of efficiency as a thickener (Tuinier *et al*., 2001).

Contrary to the role of EPS on textural properties of fermented milk, little is known about the influence of bacterial surface properties on interactions with the matrix and their functional consequences on flavour and texture (Ly‐Chatain *et al*., 2010; Jeanson *et al*., 2015; Le Boucher *et al*., 2016). More specific interactions between *L. lactis* flavours, milk proteins and emulsions were studied by Ly *et al*., 2008a,b. Here, we studied how bacterial surface properties impact textural parameters of fermented milk. For this, we used 25 isogenic *L. lactis* strains which differed in known surface properties and cell morphologies. We found that particular surface alterations lead to distinct differences in gel hardness and viscosity of fermented milk. Based on our results, we propose that bacteria can be used as structure elements in fermented foods.

## Results

### Surface‐altered Lactococcus lactis

To investigate if changes of bacterial cell surface properties affect the functionality of lactococci in fermented products, we performed assays to assess starter culture functionality in 25 isogenic *L. lactis* strains (Table 1) that differed in surface charge, hydrophobicity, chaining, clumping, attachment to proteins, pili expression and EPS production (Table 2).

**Table 1:** Strains and plasmids used in this study

No	*Lactococcus lactis* strains	Characteristic	Reference
1	NCDO712	*L. lactis* dairy isolate, *lac* ^*+*^, contains six plasmids – pLP712, pSH71, pSH72, pSH73, pSH74, pNZ712.	Gasson (1983)
2	NCDO712(pIL253*pil*)	Ery^R^; harbouring pIL253 with pilin operon *spaCB‐spaA‐srtC1‐srtC2* from NCDO712	Tarazanova *et al*. (2016)
3	MG1363	Plasmid‐cured derivative of *L. lactis* NCDO712	Gasson (1983)
4	MG1363(pIL253*pil*)	Ery^R^; *L.lactis* MG1363 harbouring pIL253 with pilin operon *spaCB‐spaA‐srtC1‐srtC2* from NCDO712	Tarazanova *et al*. (2016)
5	MG1363(pIL253*pil*∆1*)*	Ery^R^; *L.lactis* MG1363 harbouring pIL253 and pilin operon *spaCB‐spaA‐srtC1‐srtC2* from NCDO712 with 1,5 kb internal deletion in *spaCB*	Tarazanova *et al*. (2016)
6	MG1299(pIL253*pil*)	Ery^R^; *L. lactis lac* ^*+*^ derivative of NCDO712 which additionally to pLP712 harbouring the pilin operon (*spaCB‐spaA‐srtC1‐srtC2*) from the same NCDO712 strain	Tarazanova *et al*. (2016)
7	MG1299	Derivative of *L. lactis* NCDO712, harbours pLP712; *lac* ^*+*^	Gasson (1983)
8	MG1362	Derivative of *L. lactis* NCDO712 (described to harbour pSH72)	Gasson (1983)
9	MG1063	Derivative of *L. lactis* NCDO712 (described to harbour pSH73 and pSH72)	Gasson (1983)
10	MG1261	Derivative of *L. lactis* NCDO712 (described to harbour pSH73)	Gasson (1983)
11	MG1365	Derivative of *L. lactis* NCDO712 (described to harbour pSH71)	Gasson (1983)
12	MG1614	Str^R^ and Rif^R^; derivative of *L. lactis* MG1363	Gasson (1983)
13	MG1614_clu^+^	*Lac* ^*+*^. Transconjugant of MG1614, harbours pLP712 from NCDO712 and shows clumping phenotype.	Tarazanova M., Beerthuyzen M., Bachmann H., Kok J., unpublished data
14	MG1614_clu^−^	*Lac* ^*+*^. Transconjugant of MG1614, harbours pLP712 from NCDO712 and show non‐clumping phenotype.	Tarazanova M., Beerthuyzen M., Bachmann H., Kok J., unpublished data
15	MG1363∆*acmA*	Derivative of *L. lactis* MG1363 with deletion of *acmA*, which leads to chaining phenotype	Steen *et al*. (2005a)
16	MG1363∆*ahrC*	Derivative of *L. lactis* MG1363 with deletion of *ahrC*	Larsen *et al*. (2004)
17	IL1403∆*acmAacmD*	Derivative of *L. lactis* IL1403 with deletion of *acmAacmD*, which leads to chaining phenotype	Visweswaran *et al*. (2013)
18	IL1403(pIL253*pil)*	Ery^R^; Derivative of *L. lactis* IL1403 harbouring pilin operon from pSH74 of NCDO712; shows chaining phenotype and high hydrophobicity	Tarazanova *et al*. (2016)
19	MG1363∆*dltD*	Derivative of *L. lactis* MG1363 with deletion of *dltD*	Duwat *et al*. (1997), Steen *et al*. (2005b)
20	MG1363(pIL253)	Ery^R^; Derivative of *L. lactis* MG1363 harbouring plasmid pIL253	Tarazanova *et al*. (2016)
21	IL1403	Plasmid‐free derivative of *L. lactis* IL594	Bolotin *et al*. (2001)
22	MG1363(pNZ521;pIL253*pil)*	Cm^R^ and Ery^R^; derivative of *L. lactis* MG1363 harbouring pNZ521 and pIL253*pil* (*spaCB‐spaA‐srtC1‐srtC2*) from the NCDO712 strain	Tarazanova *et al*. (2016)
23	MG1363pNZ521	Cm^R^; derivative of *L. lactis* MG1363 harbouring proteolytic‐positive genes on pNZ521	Meijer and Hugenholtz (1997)
24	MG1363∆*galE*	Derivative of *L. lactis* MG1363 with deletion of *galE* leading to chain formation without galactose in a growth medium	Grossiord *et al*. (2003)
25	MG1363pNZ4120	Ery^R^; derivative of *L. lactis* MG1363 harbouring *eps* gene cluster from B40	Boels *et al*. (2003)
*Plasmids*			
1	pIL253*pil*	Ery^R^; 13.1 kb; pIL253 harbouring pSH74 pilin operon *spaCB‐spaA‐srtC1‐srtC2* with 300 bp upstream region	Tarazanova *et al*. (2016)
2	pIL253*pilΔ1*	Ery^R^; 11.6 kb; pIL253 harbouring *spaCB‐spaA‐srtC1‐srtC2* with 1.5 kb internal deletion in *spaCB*	Tarazanova *et al*. (2016)
3	pNZ521	Cm^R^; encodes the extracellular serine proteinase (PrtP) from strain SK110	Meijer and Hugenholtz (1997)
4	pLP712	The 55.39 kb plasmid encoding genes for lactose catabolism and a serine proteinase involved in casein degradation	Wegmann *et al*. (2012)
5	pIL253	Ery^R^; 4.9 kb; Low copy‐number derivative of pAMβ1	Simon and Chopin (1988)
6	pNZ4120	Em^R^; pIL253 derivative containing a 17 kb *Nco*l fragment carrying the *eps* gene cluster from NIZO B40	Boels *et al*. (2003)

**Table 2:** Phenotypic characteristics of stationary *Lactococcus lactis* strains measured at pH 6.7. Mean values ± standard deviation of biological triplicates (*n* = 3) are shown. ZP, zeta potential (mV); CSH, cell surface hydrophobicity (%); NaCN, cell attachment to sodium caseinate (%); NaCN90C, cell attachment to sodium caseinate heated at 90°C for 10 min (%); ParaCN, cell attachment to para‐caseinate (%); E24, emulsion stability after 24 h (%)

*L. lactis*	ZP, mV	CSH, %	NaCN, %	NaCN90C, %	ParaCN, %	E24,%
Pili‐overexpressing strains are chaining and clumping						
IL1403	−7.2 ± 0.5	0 ± 19.9	41.7 ± 13.6	67.2 ± 2.2	63.7 ± 1.6	0 ± 0
IL1403(pIL253*pil*)	−13.9 ± 1.3*	93.2 ± 4.5*	93 ± 2.8*	94 ± 0.4*	91.8 ± 1.8*	88.9 ± 19.3*
MG1299	−30.3 ± 2.1	75.5 ± 3.3	82.8 ± 2.7	82.7 ± 0.3	79.2 ± 2.2	35.6 ± 24.9
MG1299(pIL253*pil*)	−18.1 ± 1.3*	99.1 ± 1.2*	97.5 ± 1.1*	99 ± 0.6*	97 ± 1*	99.3 ± 0.6
MG1363pIL253	−26.4 ± 1.8	3.8 ± 6.8	99.2 ± 0.1	99.4 ± 0.3	80.6 ± 6.2	0 ± 0
MG1363(pIL253*pil*)	−11.9 ± 0.2*	94 ± 2.2*	82.8 ± 7.4	81 ± 6.8*	66 ± 16.4	85.2 ± 15*
MG1363(pNZ521)	−31.7 ± 0.8	18.7 ± 10.1	90.3 ± 2.8	88.8 ± 2.4	79.2 ± 9	0 ± 0
MG1363(pNZ521;pIL253*pil*)	−17 ± 1.9*	99 ± 1*	95.4 ± 0.2	96.5 ± 2.5	89.4 ± 3	65.6 ± 29.9*
NCDO712	−20 ± 0.5	99.4 ± 0.3	96 ± 0.6	95.7 ± 2	94.7 ± 0.6	99.7 ± 0.6
NCDO712(pIL253*pil*)	−20.5 ± 1.3	96.3 ± 0.6	85 ± 10.4	84.2 ± 9	92 ± 3.6	100 ± 0
Chaining phenotype^b^						
IL1403∆*acmAacmD*	−12 ± 0.4*	9.5 ± 4.6	97.2 ± 0.6*	96.2 ± 2.1*	96.4 ± 1.3*	0 ± 0
MG1363∆*acmA*	−31.4 ± 1.4	15.5 ± 4.5	89 ± 0.7*	87.6 ± 1	83.5 ± 10	0 ± 0
MG1363∆*galE*	−28.7 ± 0.8	14.9 ± 4.4	94.2 ± 2.1*	95.6 ± 0.5*	37.5 ± 24.6	0 ± 0
MG1363∆*dltD*	−29.2 ± 0.2	15.4 ± 3.5	84.9 ± 0.6	83.9 ± 3.1	82.4 ± 3.2	0 ± 0
Non‐chaining, non‐clumping phenotype*						
MG1363	−30.2 ± 0.7	5.8 ± 0.2	82.3 ± 0.9	82.8 ± 1.9	79.4 ± 1.7	0 ± 0
MG1261	−30 ± 1.2	21.8 ± 0.2*	93.9 ± 1.4*	92.6 ± 0.8*	88.8 ± 6.3	0 ± 0
MG1063	−29 ± 0.9	16.7 ± 0*	89.5 ± 2.6	89.6 ± 2.1	83 ± 5.1	0 ± 0
MG1362	−31.7 ± 3.3	5.6 ± 9.6	89.6 ± 3.4	84.9 ± 9.7	79.6 ± 14.1	0 ± 0
MG1365	−30.3 ± 1.2	28.6 ± 6.1*	92.9 ± 1.8*	93.1 ± 1.3*	76.6 ± 26.4	0 ± 0
MG1614	−42 ± 2.4	20.4 ± 3.1	97.1 ± 0.8	94.8 ± 4.1	97.7 ± 1.3	0 ± 0
MG1614_clu^−^	−39.4 ± 0.5	73.9 ± 4.1*	96.3 ± 0.9	94.6 ± 0.6	89.4 ± 4.9	0 ± 0
MG1363∆*ahrC*	−29.5 ± 1.2	79.9 ± 10.7*	84.5 ± 1.1	81 ± 2.7	80.2 ± 1.7	23.8 ± 2.1*
MG1363(pIL253*pil*∆1)	−16.9 ± 0.9*	79.7 ± 16.9*	66 ± 11*	64.5 ± 14.4	86.3 ± 5.2	97.8 ± 3.9*
Clumping phenotype^a^						
MG1614_clu^+^	−36 ± 0.4	90.2 ± 3.7*	81.7 ± 15	58.1 ± 26.3	94.6 ± 0.6	31.3 ± 4*
EPS producing^a^						
MG1363(pNZ4120)	−18.8 ± 2.2*	0 ± 23.8	96.2 ± 1*	98.3 ± 0.5*	97.4 ± 0.2*	0 ± 0

a Comparisons were made to *L. lactis* MG1363 except for MG1614_clu^−^/clu^+^ and MG1363(pIL253*pil*∆1) which were compared with MG1614 and MG1363pIL253 respectively.

b Comparisons were made to strains IL1403 and MG1363.

* Significant at *P* < 0.01 – comparisons were made to non‐surface modified controls.

The 25 strains are variants of the dairy isolate *L. lactis* ssp. *cremoris* NCDO712 and its plasmid and phage‐cured derivative MG1363 (Gasson, 1983). Deletion of the genes *acmA* (Steen *et al*., 2005a), *acmAacmD* (Visweswaran *et al*., 2013), *dltD* (Steen *et al*., 2005b) or *galE* (Grossiord *et al*., 2003) led to a cell chaining. The introduction of pNZ4120, a plasmid encoding the *eps* gene cluster from *L. lactis* NIZO B40 (Boels *et al*., 2003), in strain MG1363, led to EPS production, which is accompanied by a more positive surface charge (Table 2). We recently isolated strains of *L. lactis* MG1614 in which the protease/lactose plasmid pLP712 from NCDO712 was introduced through conjugation (Tarazanova M., Beerthuyzen M., Bachmann H., Kok J., unpublished data). As described earlier (Gasson and Davies, 1980), we obtained strains with clumping Clu^+^ and non‐clumping Clu^−^ phenotypes among the transconjugants carrying pLP712 (Wegmann *et al*., 2012). Surface characterization of the transconjugants showed that the non‐clumping parent strain MG1614 exhibited a hydrophobicity of ~20 ± 3%, while the hydrophobicity increased to ~74 ± 4% and ~90 ± 4% in the Clu^−^ and Clu^+^ strains respectively (Table 2).

The strains MG1365, MG1362, MG1063, MG1261 and MG1299 differ from their parent NCDO712 by carrying only one or two plasmids (Gasson, 1983). Although originally described as containing five plasmids, we recently discovered that strain NCDO712 has six plasmids (Tarazanova *et al*., 2016). One of these plasmids, pSH74, carries a *spaCB‐spaA‐srtC1‐srtC2* gene cluster, which encodes proteins that lead to pili formation on the cell surface upon overexpression (Tarazanova *et al*., 2016). The overexpression of different types of pili in *L. lactis* is known to cause auto‐aggregation (Oxaran *et al*., 2012). Our results show that overexpression of *spaCB‐spaA‐srtC1‐srtC2* leads to an increase in surface hydrophobicity from 3.8 ± 6.8% to 94 ± 2.2% and a decrease of the cell surface charge from −26.4 ± 1.8 to −11.9 ± 0.2 (Table 2). We also distinguish between chaining and clumping phenotypes. Strains exhibiting a clumping phenotype after mild centrifugation (350 g for 2 min) form strong cell aggregates that remain fast sedimenting clumps after pellet re‐suspension. Such clumping is mainly seen with pili‐overexpressing strains. In contrast, strains with a chaining phenotypes (e.g. strains carrying mutations in *acmA, dltD* or *galE*) form long chains of cocci that sediment in overnight cultures. However, after centrifugation the cell pellet can be easily re‐suspended and cells do not clump. Strain MG1363(pIL253*pil*∆1) contains the *spa* pilus gene cluster with an internal deletion of 1.5 kb in the *spaCB* gene encoding the pilin tip protein SpaC and a pilus basal subunit SpaB. This strain forms neither chains nor clumps and retained a high cell surface hydrophobicity. This strain produces pili, but they are not attached to the cell surface (Tarazanova *et al*., 2016). The used strains differ furthermore in their cell surface charge, emulsification properties and the propensity to bind milk proteins (Table 1). This morphologically diverse collection of strains was used for further analysis.

### Acidification rates

As the rate by which *L. lactis* acidifies the milk during fermentation and the final pH in the dairy product can influence the textural properties of the latter, we followed the development of pH for all strains during 21 h of milk fermentation (Table 3). It is known that very fast acidification of milk can result in excessive syneresis, whereas very slow acidification leads to the formation of a weaker gel (Gastaldi *et al*., 1997; Lucey, 2004). The maximum acidification rate for most strains was ~0.5 pH/h, but strains showing a chaining phenotype (MG1363∆*acmA,* MG1363∆*galE*), the pilin‐decorated strain MG1363(pIL253*pil*) and its control strain MG1363(pIL253) exhibited slower acidification rates (Table 3). The acidification rates within each comparison group were almost identical, leaving the surface alteration as the main variable for comparisons. The final pH of all milk samples fermented by derivatives of *L. lactis* MG1363 was 4.25 ± 0.04, with the exception of the proteolytic‐positive pilus‐harbouring strain MG1363(pNZ521;pIL253*pil*), and strain IL1403 and its derivatives (Table 3), which all exhibited elevated pH values.

**Table 3:** Textural properties of milk fermented by surface altered lactococci. Mean values ± standard deviation (*n* = 3) are shown for independent biological replications for gel hardness (g), viscosity (mPa·s) and pH

Strain	Gel hardness (g)	pH	Viscosity at shear rate of 10 s^−1^, mPa·s	Max acidification (*n* = 1), pH/h
Pili‐overexpressing strains and their parental control strains without pili (grouped per comparison)				
MG1363(pIL253)^a^	40.1 ± 2.18	4.29 ± 0.05	722.7 ± 11.3	−0.36
MG1363(pIL253*pil∆1*)^a^	45.1 ± 1.13*	4.23 ± 0	1050 ± 19.1*	−0.39
MG1363(pIL253*pil*)^a^	47.8 ± 1.8*	4.23 ± 0.05	1079.3 ± 58.2*	−0.39
MG1363(pNZ521)^a^	42.8 ± 0.4	4.22 ± 0.05	773 ± 64.3	−0.49
MG1363(pNZ521; pIL253*pil*)^a^	49.1 ± 2.8*	4.34 ± 0.006	1027.2 ± 30*	−0.46
NCDO712^a^	43.5 ± 0.6	4.24 ± 0.06	1498 ± 60.6	−0.39
NCDO712(pIL253*pil*)^a^	42.4 ± 3.2	4.23 ± 0.006	1192 ± 146.2*	−0.34
MG1299	38.59 ± 0.6	4.23 ± 0.05	726.1 ± 68.1	−0.49
MG1299(pIL253*pil*)	38.2 ± 0.6	4.27 ± 0.001	797 ± 54	−0.54
IL1403	37.5 ± 0.64	4.45 ± 0.02	680.1 ± 34.5	−0.53
IL1403(pIL253*pil*)	37 ± 0.7	4.47 ± 0.001	745 ± 78	−0.51
Combinations of EPS and pili/no‐pili forming strains				
MG1363(pNZ4120)^a^	48.9 ± 1.9	4.32 ± 0.006	4556 ± 37.7*	−0.53
MG1363(pNZ4120) + MG1363(pIL253*pil*)*	51.9 ± 1.1	4.22 ± 0.006	3231 ± 78.9	NA
MG1363(pNZ4120) + MG1363(pIL253*pil∆1*)	44.4 ± 3.6	4.19 ± 0.006	2921 ± 277.7	NA
MG1363(pNZ4120) + MG1363(pIL253)^a^	46.9 ± 2.9	4.24 ± 0.006	3242.7 ± 396.7	NA
MG1363(pNZ4120) + NCDO712	45.6 ± 6.8	4.18 ± 0.01	2847 ± 117.1	NA
MG1363(pNZ4120) + NCDO712(pIL253*pil*)	46.97 ± 1.48	4.21 ± 0	2976 ± 105.5	NA
Chaining strains and their parental controls				
IL1403	37.5 ± 0.64	4.45 ± 0.02	680.1 ± 34.5	−0.53
IL1403∆*acmAacmD*	35.4 ± 0.14*	4.39 ± 0.01	1008.5 ± 109.3*	−0.51
MG1363	43.78 ± 0.89	4.24 ± 0.05	926.1 ± 50.5	−0.53
MG1363∆*acmA*	41.5 ± 1.08*	4.24 ± 0.05	1034.7 ± 18.9*	−0.42
Transconjugants (clumping/non‐clumping)				
MG1614	40 ± 0.43	4.23 ± 0.05	832.5 ± 40.9	−0.54
MG1614_clu^−^	39 ± 1.19	4.27 ± 0.06	739 ± 37*	−0.5
MG1614_clu^+^	41.7 ± 1	4.27 ± 0.05	887.9 ± 28	−0.5
NCDO712 derivatives carrying one or two plasmids and MG1363 derivatives with single gene deletions				
NCDO712	42.4 ± 1.3	4.24 ± 0.06	867.2 ± 106.1	−0.51
MG1363	43.78 ± 0.89	4.24 ± 0.05	926.1 ± 50.5	−0.53
MG1363∆*ahrC*	43.8 ± 0.43	4.28 ± 0.03	771 ± 205.5	−0.56
MG1363∆*dltD*	43.9 ± 0.56	4.24 ± 0.05	891.9 ± 66.9	−0.52
MG1363∆*galE*	44.71 ± 0.58	4.21 ± 0.05	871.1 ± 181.3	−0.45
MG1063	45.17 ± 0.39	4.19 ± 0.05	986.97 ± 20.7	−0.53
MG1261	41 ± 0.91	4.21 ± 0.05	790.97 ± 20.6	−0.54
MG1362	44.1 ± 0.81	4.2 ± 0.04	945.1 ± 40.9	−0.52
MG1299	38.59 ± 0.6	4.23 ± 0.05	726.1 ± 68.1	−0.49

NA, not applicable. For cell mixtures, the acidification rate was not determined.

a For the indicated strains independent repeats of the experiments on different days confirmed the results with slightly different absolute values.

* Significant at *P* < 0.05 – comparisons were made to non‐surface modified controls as described in Table 2.

### Gel hardness

To determine if cell surface properties influence the rheological parameters of fermented milk, we measured gel hardness and viscosity. The gel hardness of fermented milk prepared with the pili‐overexpressing strain MG1363 (pIL253*pil*) increased by approximately 46% (*P* = 0.009) compared with milk fermented with the empty plasmid control strain MG1363(pIL253) (Table 3). Similar to MG1363(pIL253*pil*), the proteolytic‐positive variant overexpressing pili, strain MG1363(pNZ521; pIL253*pil*), also increased the fermented milk gel strength, by 15% (*P* = 0.04). For three other strains, NCDO712(pIL253*pil*), MG1299(pIL253*pil*) and IL1403(pIL253*pil*), gel hardness did not change significantly compared with their control strains. The fact that IL1403(pIL253*pil*) did not change gel strength can be explained by the very low number of pili on the surface of the cells as compared with MG1363(pIL253*pil*) (Tarazanova *et al*., 2016). The data suggest that the overexpression of pili has a profound effect on gel hardness, but the effect seems to be strain‐dependent. When chaining or clumping cells were used gel hardness was significantly decreased. The chain‐forming strains IL1403∆a*cmAacmD* and MG1363∆*acmA* decreased gel hardness compared with their controls by 5.65% (*P* = 0.005) and 5.2% (*P* = 0.049) respectively. Together, these results show that microbial cell surface alterations brought about by either pili overexpression or cell chaining significantly alters the gel hardness of fermented milk.

### Viscosity

Viscosity was increased by 19–35% for milk fermented with the strains overexpressing pili, MG1363(pIL253*pil*) (*P* = 0.0005) and MG1363(pNZ521; pIL253*pil*) (*P* = 0.003), relative to their controls (Table 3). In comparison to milk fermented with the EPS‐producing strain MG1363pNZ4120 (392% increase compared with MG1363, *P* = 0.0007) the increase in viscosity caused by pili production is considerably lower. We did not observe synergistic effects when milk was fermented with a 1:1 mixture of EPS‐ and pili‐producing strains (Table 3).

The use of chaining phenotypes lead to milk gels with increased viscosity. For example, the chaining strains MG1363∆*acmA* and IL1403∆*acmAacmD* formed fermented milk with a viscosity that was 11% (*P* = 0.025) and ≈ 50% (*P* = 0.026) higher compared with the control strains MG1363 and IL1403 respectively (Table 3). Overall, the results of milk viscosity measurements show that the engineering of the surface morphology of *L. lactis* increases product viscosity by up to 50%.

### Localization of cells in fermented milk

**Figure 1:**
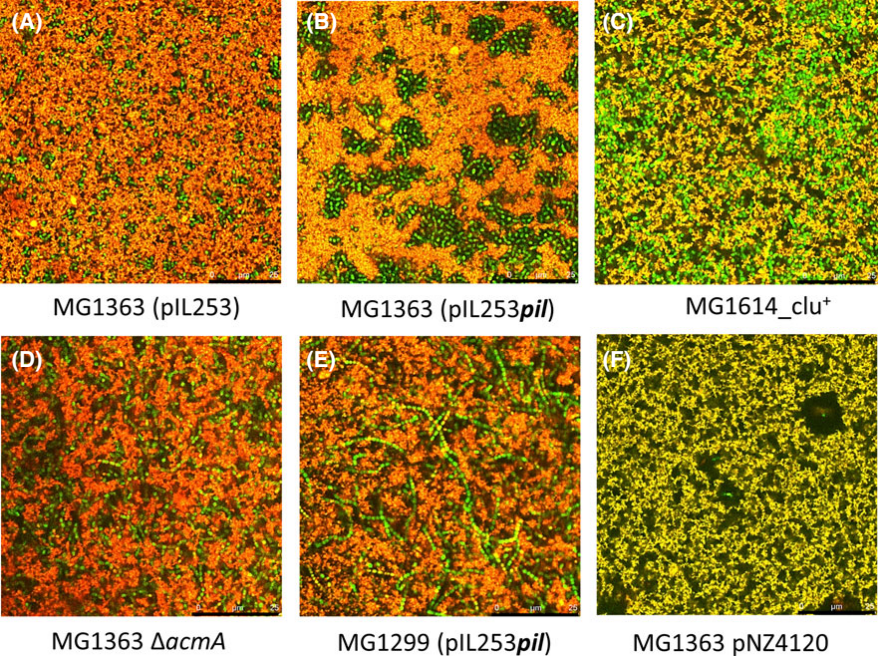
Microstructure of milk fermented by *Lactococcus lactis* strains with altered surface properties. Bacterial cells are green, while proteins and lipids appear orange/red; the black areas represent the serum fraction. The black bar in the right corner indicates 25 μm.

To investigate if alterations of the bacterial cell surface affect the location of cells in the fermented milk matrix, we performed CLSM imaging on undisturbed fermented milk samples. Due to the sedimentation of cells during fermentation and the limited depth at which CLSM allows imaging (~ 40 μm), the number of cells in the image might be higher than in the average sample. Nevertheless, we consider the observed effects to be indicative for what occurs throughout the product and the CLSM images to provide information on the behaviour of the bacteria *in situ*. The results (Fig. 1) reveal obvious differences between various strains. For example, loose cocci and clumping cocci locate in the protein matrix close to serum regions (Fig. 1A and C), aggregated cells of MG1363(pIL253*pil*) fill the serum regions between the aggregated proteins in the fermented milk (Fig. 1B), chaining cells are located in protein and serum regions (Fig. 1D and E), while EPS‐producing cells locate predominantly in serum regions (Fig. 1F). Interestingly, the localization effect observed for MG1363(pIL253*pil*) was not seen for strain IL1403(pIL253*pil*). This would be consistent with the fact that pilin overexpression results in much fewer pili on the surface of IL1403 as compared with MG1363 (Tarazanova *et al*., 2016) and the limited effect on gel hardness presented above. Overall the CLSM results indicate that surface properties of *L. lactis* can have profound effects on the location of the cells in the fermented milk matrix.

## Discussion

Important physical properties of fermented milk are gel hardness and viscosity. It is known that the hardness of fermented milk increases with a higher dosage of casein (Oliveira *et al*., 2001), upon heat treatment (Lucey *et al*., 1997) or homogenization of milk (Serra *et al*., 2009), decreasing pH (Schkoda *et al*., 1999), longer acidification time or rate (Kristo *et al*., 2003) or lower post‐incubation temperature (Lucey and Singh, 1997). The viscosity of fermented milk increases when the gel is allowed to harden before stirring, when stirring intensity is lowered or when the bacterial strains applied produce EPS (Girard and Schaffer‐Lequart, 2007). Here we investigated the potential role of bacterial surface properties and morphology on the textural and structural parameters of fermented milk. Comparisons were made between engineered strains showing alterations in cell chaining, clumping, exopolysaccharide production and pili formation and their isogenic parental strain.

We found that the alterations of cell surface morphology are accompanied by changes in surface charge and hydrophobicity. The bacterial surface properties of these strains also affected the viscosity and gel hardness of milk fermented with them. In addition, the bacterial localization in the fermented milk matrix (Table 4) was dependent on cell surface morphology.

**Table 4:** Summary of textural parameters of milk fermented with surface‐altered lactococci

Phenotype	Strain^a^	Viscosity, %	Gel hardness, %
Pili overexpression	MG1363(pIL253*pil*)	+49.3**	+19.2**
	MG1363(pIL253*pil*∆1)	+45.3*	+12.5*
	MG1363(pNZ521; pIL253*pil*)	+40.1**	+14.7*
	MG1299(pIL253*pil*)	+9.8	−1
	NCDO712(pIL253*pil*)	−20.4*	−2.5
	IL1403(pIL253*pil*)	+9.5	−1.3
Chaining	IL1403∆*acmAacmD*	+48.3*	−5.6**
	MG1363∆*acmA*	+11.7*	−5.2*
Clumping	MG1614_clu^+^	+6.7	+4.3

a All comparisons are made with the isogenic parent strain.

* Significance (*P* < 0.05).

** Significance (*P* < 0.01).

The bacterial cell surface contains charged hydrophilic as well as hydrophobic areas, which are the result of the complex molecular composition of the cell wall. For example, overexpression of pili in MG1363(pIL253*pil*) led, in neutral pH, to a decrease in charge (from −26 ± 2 mV to −12 ± 0 mV). The decrease in net‐negative charge can possibly be explained by the slightly net‐positive charge of the overexpressed pilin proteins. The charged residues of pilin proteins are likely to be on the outside of the pilus (Tsumoto *et al*., 2004). Analysis of the *spa* pilin protein sequences (http://www.expasy.org/) showed 30–49% hydrophobic residues. The increase in hydrophobicity from 5% to 96% of MG1363(pIL253*pil*) relative to its parent might be explained by hydrophobic patches on the overexpressed pili. Taken together bacterial amphiphilic surface properties are governed by the molecular composition of its cell wall, which is strongly strain‐dependent (Tarazanova *et al*., 2017) and can be engineered.

The increased viscosity and gel hardness of fermented milk using pili overexpression strains was seen independently for three out of the six strains tested, which indicates that this effect is strain‐dependent. The three strains where this effect was not observed are NCDO712, MG1299 and IL1403. Strain NCDO712 carries the *spa* pilin genes on the low copy‐number plasmid pSH74, which are not expressed at high levels (Tarazanova *et al*., 2016). Similarly, electron microscopy revealed that IL1403(pIL253*pil*) carried low numbers of pili on the surface (Tarazanova *et al*., 2016). Strain MG1299(pIL253*pil*) forming long chains shows a trend towards higher viscosity but it has no effect on gel hardness. This phenomenon is in agreement with our observations for solely chaining strains described below. The 20–35% increase in viscosity obtained with the other strains is a substantial alteration that is likely to be perceived sensorically.

The effect of the microbial surface on textural properties of fermented milk leads us to hypothesize that bacteria can be seen and used as structural or ingredient elements of a food matrix. As such they engage in physico‐chemical interactions with milk proteins and/or with other cells via molecules on their polymeric cell surface layers (Ubbink and Schär‐Zammaretti, 2005; Jacobs *et al*., 2007; Burgain *et al*., 2013). The occurrence of interactions is a balance between attractive and repulsive forces, which strongly depend on the interacting particles. For instance, it was shown that for *Lactobacillus bulgaricus* GG the force between micellar casein (or denatured whey protein) and the bacterial cell is about 0.4 nN, but this was significantly different for other strains (Burgain *et al*., 2014b). Together with our results, these observations suggest that the type and force of microbe–matrix interactions can influence the structure, stability and textural properties of the fermented milk matrix.

Strains of *L. lactis* show a high degree of biodiversity in surface properties among which their capacity to bind to milk proteins. We have previously reported that dairy isolates display a higher milk protein binding affinity than plant isolates (Tarazanova *et al*., 2017). The increased viscosity and gel hardness of fermented milk might, thus, also be caused by bacterial interactions with milk proteins (Tarazanova *et al*., 2017) as well as by cell to cell connections via pili between the starter culture cells when they are in a close proximity (Tarazanova *et al*., 2016). The results of the current study show that despite the surface alterations inflicted, the lactococcal cells retained a high capacity to bind to milk proteins (Table 2). These results indicate that factors other than pili or morphology changes are responsible for milk protein binding.

Interactions of *L. lactis* with milk proteins might result in preferential localization of the bacterial cells in the protein matrix or in serum regions, in the form of aggregates and/or chains. This is corroborated by microscopic observations of pili‐overexpressing cells in the milk matrix. These cells seem to be localized in the serum regions (Fig. 1). Here the size and shape of particles is of importance. Cells do not only form very strong cell–cell connections, leading to cell aggregates located in milk gel cavities, but also cell–protein contacts between pili of outer cells of aggregates and proteins of milk gel, which appeared to contribute to the increased milk gel hardness.

Similar to pili‐expressing cells, which show a chaining and, after centrifugation, a clumping phenotype, bacteria that only form chains (*acmA*/*acmD* deletions) increased the viscosity up to 12–48%, and in contrast to pili‐expressing cells this lead to a decrease in gel hardness by 6–14% (Tables 2 and 3). It was detected that there are more cavities in milk gel fermented with chaining strains. The decreased gel hardness can be explained by cavities of serum in the milk matrix: they do not provide any additional bonds to strengthen the gel. We speculate that that the cells do not interact with the aggregated casein micelles in this case and are, thus, not included in the matrix but act like structure breakers or inert fillers. This would be similar to what is seen for fat droplets with natural milk fat globular membranes in milk gels made with unhomogenized milk (Lucey and Singh, 1997): in this case, the matrix has to form around those droplets. Also longer cell chains can be considered as “viscosifying” molecules and not as enhancing properties for the gel hardness. Chain length might be at the basis of the increased viscosity of a matrix obtained by fermenting milk with chaining cells. In general, the longer the molecule (especially when it is stretched) the more viscosifying properties it has due to possible hydrogen bonding with water molecules as well as molecule–molecule interactions or entanglements.

The findings presented here show that engineering surface properties of dairy starter strains allows altering product properties such as gel hardness and milk viscosity. It will be highly interesting to see whether the quality changes can be perceived in sensory analyses. This opens possibilities to develop new concepts in improving fermented products with altered textural properties.

## Experimental procedures

### Bacterial strains and growth conditions

*Lactococcus lactis* strains (Table 1) were grown at 30°C in M17 broth (Oxoid Ltd, Basingstoke, UK) containing 1% glucose (GM17) or 1% lactose (LM17). When required, erythromycin (Ery; 10 μg ml^−1^), chloramphenicol (Cm; 5 μg ml^−1^), rifampicin (Rif; 50 μg ml^−1^) or streptomycin (Str; 100 μg ml^−1^) were added to the indicated final concentrations.

### Cell surface properties

Bacterial cell surface properties, i.e. surface charge (or zeta potential, ZP, mV), cell surface hydrophobicity (CSH, %), emulsion stability after 24 h (E24, %) and attachment to milk proteins were measured as described earlier (Tarazanova *et al*., 2017).

### Preparation of fermented milk

Full‐fat pasteurized and homogenized milk (3.6% fat, 3.5% protein, 4.7% lactose) was purchased from a local supplier and sterilized at 115°C for 15 min to denature the whey proteins (Sodini *et al*., 2004). Sterilized milk was supplemented with sterile 50% glucose solution (4% final concentration) and sterile 20% Bacto^™^ Casitone (Pancreatic digest of casein, BD, Sparks, MD, USA) solution to 0.2% final concentration to ensure growth of lactose‐negative and protease‐negative strains respectively. Instead, for lactose‐positive and protease‐positive strains, a same volume of water was added to the milk to ensure that protein and fat content of the milk were the same for all strains. For gel strength and viscosity measurements on the fermented milk samples, the bacterial strains were pre‐cultured overnight in milk supplemented with the appropriate antibiotic, but the antibiotic was not added during the actual milk fermentation.

Warm (30°C) milk was inoculated with 2% v/v of an overnight culture. Acidification rates and final pH were measured with a Cinac 14 ph+2T (Alliance instruments, Freppilon, France). pH electrodes were inserted into the inoculated milk samples in 10 ml tubes and measurement was done every 6 min for 21 h at 30°C.

### Gel strength of fermented milk

Aliquots (300 ml) of milk without antibiotic were inoculated with 2% of the strains to be tested. The prepared milk was distributed to three 100 ml sterile glass cups (70 mm diameter), which were incubated statically for 21 h at 30°C. Gel strength was measured with a Texture Analyzer (TA.XTplus, Stable Micro Systems Ltd., Sprundel, NL) equipped with a 5 kg load cell according to the manufacture application guidelines. The 100 ml fermented milk samples were compressed uniaxially to a depth of 20 mm with a constant speed of 1 mm s^−1^ by a probe with a grid‐like geometry having 10 mm side squared openings. The peak force applied on the sample corresponds to the hardness of the milk gel.

### Viscosity of fermented milk

After the texture analysis, the viscosity of the fermented milk was measured with a rotational viscometer (Haake Searle RV20 Rotovisco and RC 20 Rheocontroller, ThermoScientific, Hofheim, Germany) with MV2P (medium viscous profile) rotor according to the manufacture guidelines. Fermented milk (60 g) was transferred to the MVP cup and allowed to rest for 15 min. Subsequently, the sample was measured as the shear rate gradually increased from 0 to 400 s^−1^ for 5 min and then decreased again from 400 to 0 s^−1^ for 5 min at 21°C. Viscosity values were determined at a shear rate of 10 s^−1^ of the first curve, i.e. when shear rate was increasing.

### Confocal laser scanning microscopy (CLSM)

To 1 ml of homogenized sterilized milk, with sugar and antibiotics as required, 15 μl of a solution containing 0.5% (final concentration) Acridine Orange (Sigma‐Aldrich Chemie B.V., Zwijndrecht, The Netherlands) and 0.025% (final concentration) Rhodamine B (Sigma‐Aldrich Chemie B.V., Zwijndrecht, The Netherlands) in water was added to stain proteins and fat droplets respectively. This milk was inoculated with 1% of an overnight culture, after which 1 μl Syto 9 (5 mM in DMSO, ThermoFisher; 1 μl/OD_600_ of 1/ml) was immediately added to the sample to stain the bacterial cells. The milk sample was subsequently transferred to a CLSM slide with a cylindrical plastic cup attached to it which was then covered with a lid (Hassan *et al*., 1995) to prevent evaporation. The sample was incubated for 21 h at 30°C.

CLSM images were taken using a Leica TCS SP 5 confocal laser scanning microscope (Leica Microsystems CMS GmbH, Mannheim, Germany) with Leica application Suite Advanced Fluorescence v. 2.7.3. build. 9723. The Argon laser was used to visualize the Syto9‐stained bacteria, while the DPSS 561 laser was used to visualize the milk protein and fat droplet in the matrix that were stained by the Acridine Orange and Rhodamine B respectively. The objective lens used was an HCX PL APO 63 × /1.2/water CORR CS.

## Conflict of interests

The project was funded by TiFN, a public–private partnership on precompetitive research in food and nutrition. The public partners are responsible for the study design, data collection and analysis, decision to publish and preparation of the manuscript. The private partners have contributed to the project through regular discussion. A patent application pertaining to the presented findings was filed.
